# The impact of a community intervention to improve cervical cancer screening uptake in the Amazon region of Brazil

**DOI:** 10.1590/S1516-31802007000100008

**Published:** 2007-01-04

**Authors:** Marcus Vinicius Von Zuben, Sophie Françoise Derchain, Luis Otávio Sarian, Maria Cristina Westin, Luiz Claudio Santos Thuler, Luiz Carlos Zeferino

**Keywords:** Cervix uteri, Neoplasms, Diagnosis, Ambulatory care, Vaginal smears, Colo uterino, Câncer, Diagnóstico, Assistência ambulatorial, Esfregaço vaginal

## Abstract

**CONTEXT AND OBJECTIVE:**

In the northern region of Brazil, cervical cancer is the most important cause of cancer-related deaths among women. There is considerable likelihood, however, that official incidence and mortality figures are greatly underestimated. The aim of this study was to estimate the repercussions from improvement in cervical cancer screening programs on the incidence of pre-invasive and invasive cervical lesions in a municipality in this region.

**DESIGN AND SETTING:**

This was a quasi-experimental study that assessed process dimensions relevant to the program objectives. The study comprised a sample of 2,226 women seen at primary healthcare units in Cruzeiro do Sul, a small city in the Brazilian Amazon region, from April 2003 to July 2004.

**METHODS:**

Women were recruited through local radio advertisements and by oral communication from the investigators. The women answered a structured questionnaire and underwent pelvic examination, which included Papanicolaou (Pap) smears and naked-eye inspection of the cervix after applying diluted acetic acid. Women with positive Pap smears or abnormal gynecological examination were referred for colposcopy and possible biopsy, diathermic large loop excision of the transformation zone or conization.

**RESULTS:**

The results obtained were compared with historical official data retrieved from the Brazilian Ministry of Health's database. Intervention resulted in a 40% increase in positive Pap smears and detection of cancer was nine times higher than had been observed in routine screening.

**CONCLUSIONS:**

Detection of pre-invasive and invasive cervical lesions in the intervention group was remarkably higher than among women seen during routine screening.

## RESUMO

**CONTEXTO E OBJETIVO:**

Na Região Norte do Brasil, o câncer do colo do útero é a mais importante causa de morte, por câncer, entre as mulheres. É muito provável, entretanto, que os dados oficiais referentes à incidência e à mortalidade estejam grosseiramente subestimados. O objetivo foi estimar as repercussões que a melhora dos programas de rastreamento possam exercer sobre a incidência de lesões pré-invasoras e invasoras do colo do útero em uma municipalidade da região Amazônica brasileira.

**TIPO DE ESTUDO E LOCAL:**

Este estudo “quasi-experimental” avaliou dimensões processuais relevantes para os objetivos do programa de rastreamento. Foi incluída uma amostra de 2.226 mulheres, atendidas em unidades básicas de saúde de Cruzeiro do Sul, uma pequena cidade da Amazônia Brasileira, entre abril de 2003 e julho de 2004.

**MÉTODOS:**

As mulheres foram recrutadas através de anúncio pelo rádio e por comunicação oral desempenhada pelos próprios pesquisadores. Após assinarem o Termo de Consentimento Informado, responderam a um questionário estruturado e foram submetidas a exame pélvico, o que incluiu coleta e colpocitologia oncológica (CO) e inspeção da cérvice após aplicação de ácido acético diluído. Mulheres com CO positiva ou exame ginecológico anormal foram encaminhadas para colposcopia e eventual biópsia, excisão diatérmica da zona de transformação ou conização.

**RESULTADOS:**

Os resultados obtidos foram comparados a dados históricos oficiais referentes à cidade de Cruzeiro do Sul, recuperados do banco de dados do Ministério da Saúde do Brasil. Em comparação aos dados históricos, a intervenção resultou em um aumento de 40% na freqüência de CO positiva, e a detecção de câncer foi nove vezes superior àquela dos dados oficiais.

**CONCLUSÕES:**

A detecção de lesões cervicais pré-invasoras e invasoras no grupo que sofreu intervenção foi marcadamente superior à das mulheres atendidas pelo programa oficial de rastreamento.

## INTRODUCTION

Cervical cancer is the second most common neoplasia among women worldwide. The disease burden is higher in the developing world, and it has been estimated that, in the northern region of Brazil, it is the most important cause of cancer-related deaths among women (4.6 per 100,000 women), with an incidence of 16.75 cases per 100,000 women for the year 2006.^1^ Specifically for the State of Acre, in the Brazilian Amazon region and one of the smallest states in Brazil, with only 670,000 residents, the mortality rate due to cervical cancer was 4.02 and there were 14.53 new cases for each 100,000 women in 2002.^1,2^ There is a considerable likelihood, however, that these already high figures were greatly underestimated. It is suspected that many cases are inadequately recorded, because the population cannot access health services and their social and economic circumstances are very unfavorable.

Cervical cancer screening programs are based on the detection of pre-invasive lesions, i.e. cervical intraepithelial neoplasia (CIN). Cervical cytological tests (Papanicolaou tests, or Pap smears) are the primary screening tool used in Brazil. However, in order to be effective, cytology-based screening programs necessitate a complex and expensive infrastructure: primary healthcare units to systematically collect the smears, trained professionals, laboratories for slide processing and reading and, finally, professionals capable of dealing with the abnormalities that may be detected.

## OBJECTIVE

The purpose of the present study was to estimate the changes in the incidence of pre-invasive and invasive cervical neoplasia that a possible improvement in access to health services would cause, in a small and isolated province of the Amazon region, in northern Brazil.

## METHODS

### Study site

This study was carried out in the city of Cruzeiro do Sul, which is located in the extreme west of the Brazilian Amazon basin, in the State of Acre. It has been estimated that, in 2001, the municipality had 67,441 inhabitants,^3^ 52% of them living in rural areas. The city is accessible mainly by river or air transportation, because during the rainy season the main roads to the city remain unserviceable. Illiteracy is very common: approximately 40% of the population cannot read or even sign their own names.^2^ Economic development is negatively affected by the region's geographical position, in the middle of the Amazon forest, and by several concurrent social problems.

### Community-level intervention

Women were recruited through local advertisements, by radio broadcasting and oral communication; the latter was performed by the investigators themselves. To be eligible to enter the study, women had to: a) provide informed consent by signing (or placing their fingerprints on) the informed consent form (which was read out by one of the investigators when the women were unable to read it themselves); b) have a cervix uteri; c) be outside of the bleeding period of the menstrual cycle; d) have not had sexual intercourse or applied vaginal creams over the three days prior to consultations. Women in this last condition were advised to schedule a new appointment in a few days thereafter. Women were excluded if they: a) had undertaken a Pap test during the last year prior to this consultation; b) had undertaken cervical cauterization, biopsy or conization during the last year prior to this consultation; c) were pregnant; d) had had cervical neoplasia (any type) in the past.

A total of 2,226 women were enrolled, and consultations were carried out in the primary healthcare units of Cruzeiro do Sul, from April 2003 to July 2004. In addition to the consultations performed for the purposes of this study, another 3,780 routine Pap tests were carried out in Cruzeiro do Sul while the study was underway.

### Consultion Routine

After signing the informed consent form, the women responded to a structured questionnaire and were subjected to pelvic examination, with inspection of the cervix and collection of samples for the Pap test. As a final step, 3.0% diluted acetic acid was applied to the cervix, and the cervix was re-examined using the naked eye. Abnormal results from the cervical inspection or Pap test prompted referral for colposcopy, with possible biopsy or diathermic excision of the transformation zone, depending on the characteristics of the lesion.

### Pap smear

Pap smears were taken using an Ayre spatula and endocervical brush. They were fixed in 95% ethanol and stained using the modified Papanicolaou method. The final cytological diagnosis was obtained using the Bethesda System,^4^ and the results were classified as normal/inflammatory, atypical squamous cells (ASC), atypical glandular cells (AGC), low-grade squamous intraepithelial lesion (LSIL), high-grade squamous intraepithelial neoplasia (HSIL) or suggestive of cancer. The cytological slides from smears collected from patients enrolled to this study were read by an experienced cytopathologist, based at the cytology laboratory of Universidade Estadual de Campinas. On the other hand, patients screened normally, within the official program, had their Pap tests performed by local laboratories in the State of Acre. For statistical purposes, normal/inflammatory results were categorized as negative, and ASC, LSIL or HSIL as positive.

### Histolog

Biopsy and conization specimens were fixed in 10% phosphate-buffered formalin, embedded in paraffin, and stained with hematoxylin and eosin (HE). Biopsies were analyzed according to the World Health Organization (WHO) criteria^5^ and classified as negative, CIN 1, CIN 2, CIN 3 or invasive squamous carcinoma.

### Statistical methods

Box plot representations of the mean, median and standard deviations of age at diagnosis for each pre-neoplastic or neoplastic lesion were produced. The performance estimators, or the percentages of positive screening tests across different categorical strata, were compared with historical data retrieved from the Brazilian Ministry of Health's database.^1^ The cancer detection rate was calculated per 1,000 women screened.

## RESULTS

The patients’ ages ranged from 20 to 83 years (mean: 39.6 years). Of the 2,226 Pap smears collected, 2,106 (94.6%) were classified as negative. The most frequent Pap abnormality was ASC (1.9%), followed by HSIL (1.8%), LSIL (0.7%), AGC (0.6%), squamous cells carcinoma (0.3%) and adenocarcinoma *in situ* (0.09%).

Cervical inspection revealed abnormalities in 21.2% of the women, and 541 women were examined by means of colposcopy. Abnormal findings from colposcopy were found in 26% of the women that had undertaken the examination, and 55.8% of these women also had a positive Pap test (data not shown).

**Figure 1:**
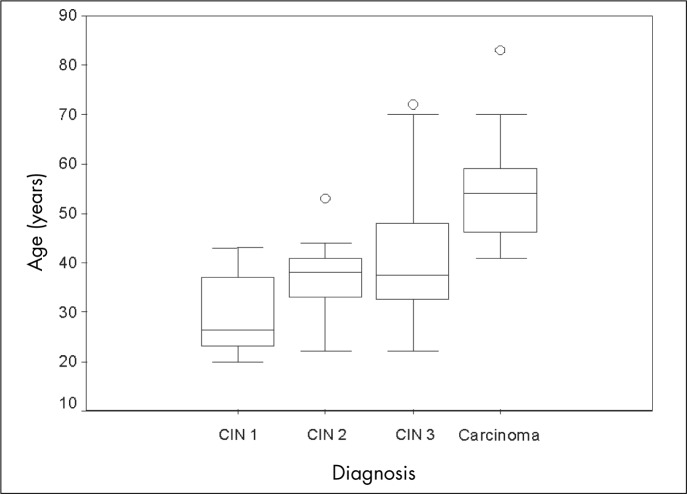
Age-related distribution of final diagnoses of cervical intraepithelial neoplasia (CIN) in 2,226 women examined in a municipality in the Amazon region

Visually detectable lesions were found in a few women with negative cytology: six cases of CIN 1, ten of CIN 2 or 3 and one case of cancer. The majority (78.6%) of the women with AGC were found to be free of histologically-confirmed disease, and the same occurred in 63.6% of the women with ASC. Three (6.8%) of the women with ASC had cancer, and 13 (29.6%) of these were diagnosed with CIN 2 or 3. Among women with LSIL, six (37.5%) were diagnosed with CIN 2 or 3. The proportion of women with histologically-confirmed disease was even higher among women with HSIL, since 81.1% of them had CIN 2 or worse. All the women with Pap smears suggestive of cancer did indeed have high-grade disease (Table 1). The majority (69.4%) of the CIN cases occurred in women below 40 years of age, and disease severity increased in parallel with age (Figure 1).

**Table 1.** Final diagnoses from the cytology results of 2,226 women screened for cervical cancer

Histology	Cytology							
	Neg	AGC	ASC	LSIL	HSIL	*In situ* adenocarcinoma	Invasive adenocarcinoma	Total
	n (%)	n (%)	n (%)	n (%)	n (%)	n (%)	n (%)	n (%)
Neoplasia absent	2,089 (91.2)	11 (78.6)	28 (63.6)	7 (43.8)	7 (18.9)	0	0	2,142 (96.2)
CIN 1	6 (0.28)	0	0	3 (18.8)	0	0	0	9 (0.4)
CIN 2	3 (0.14)	0	4 (9.1)	4 (25.0)	4 (10.8)	0	0	15 (0.7)
CIN 3	7 (0.33)	3 (21.4)	9 (20.5)	2 (12.5)	24 (64.9)	0	3 (42.9)	48 (2.2)
*In situ* adenocarcinoma	0	0	0	0	1 (2.7)	1 (50.0)	0	2 (0.09)
Invasive squamous carcinoma	1 (0.05)	0	3 (6.8)	0	1 (2.7)	0	4 (57.1)	9 (0.4)
Invasive adenocarcinoma	0	0	0	0	0	1 (50.0)	0	1 (0.04)
**Total**	**2,106 (100.0)**	**14 (100.0)**	**44 (100.0)**	**16 (100.0)**	**37 (100.0)**	**2 (100.0)**	**7 (100.0)**	**2,226 (100.0)**

*Neg = negative; AGC = atypical glandular cells; ASC = atypical squamous cells; LSIL = low-grade squamous intraepithelial lesion; HSIL = high-grade squamous intraepithelial neoplasia; CIN = cervical intraepithelial neoplasia.*

The proportion of cytology abnormalities in this series was 40% higher (ratio: 1.40) than what has been officially reported for the city of Cruzeiro do Sul.^3^ However, during the intervention period of the study, the number of HSIL or worse Pap tests was four times higher (ratio: 4.1) than the official historical figures, and the proportion of cancer cases detected in the population was nine times higher (ratio: 9.3) than what was observed from routine screening (Table 2).

**Table 2.** Screening performance estimators for cervical cancer

	Historical data^1^ (2001-2002)	This study (2003-2004)	Ratio
Number of tests in target population	2993	2226	—
Percentage of screening tests with positive results	3.9	5.4	1.4
Percentage of screening tests positive for HSIL or cancer	0.5	2.1	4.1
• Percentage of screening tests positive for HSIL	0.5	1.7	3.5
• Percentage of screening tests positive for cancer	0.03	0.3	9.3
Cancer detection rate per 1,000 women screened (final diagnosis)	NA	4.49	---

*NA = not available; HSIL = high-grade squamous intraepithelial neoplasia.*

## DISCUSSION

The primary purpose of this study was to evaluate the consequences of a community-based intervention for the screening of women who had not had a Pap test over the preceding 12 months, in a remote municipality in the Amazon region. In this intervention study, in comparison with the official historical data and routine screening in Cruzeiro do Sul, a remarkably higher number of abnormal Pap tests were found and many more cases of cancer were detected. It should be noted, however, that invasive cancer detection rates generally exceed the cancer incidence rates in the population, because some cancers would remain asymptomatic in the absence of screening. In the present series, the cancer detection rate (4.49 cases per 1,000 women screened) was 30 times higher than the officially estimated incidence of the disease in Cruzeiro do Sul (14.53 per 100,000 women).^3^

In countries where the female population is screened appropriately, it has been estimated that less than 1% of Pap smears should harbor a cytological abnormality.^4,6^ In the present study, 5.4% of the Pap smears were considered to be abnormal. This can be attributed to inadequate previous screening (if any), and to the criterion of only including women who had not had a Pap test during the 12 months prior to enrollment. In addition, the personnel involved in the study were well-trained and performed rigorous quality control at all stages of the screening, from recruiting the women to their treatment.

In alignment with the high rate of Pap abnormalities found in this study, 21.2% of the women also had abnormalities in their cervix that were visible to the naked eye. In India and some African nations, investigators have analyzed the possible role of visual inspection of the cervix with acetic acid (VIA), alone or in combination with Pap smears, as a screening tool. In one of these studies, reporting on 2,754 women, 29.4% of the subjects aged 35-39 years old had abnormal findings from VIA, while this rate decreased to 23.4% in women aged 50-65 years.^7-9^

High sensitivity is probably the most desirable feature in a screening test. The sensitivity of Pap smears, when the test is performed under ideal conditions (i.e. adequate collection, slides immediately fixed and experienced cytologists) barely exceeds 70%.^10,11^ On the other hand, it is also desirable that the number of false-positive results be reduced to a minimum, thereby avoiding unnecessary referrals for colposcopy. It has been well established that colposcopy places a heavy burden on healthcare systems, because it can only be performed by trained physicians, thus restricting its usage in underdeveloped regions. In the present study, abnormal colposcopy findings were present in 55.8% of the women who had altered Pap smears. However, it has to be mentioned that, in contrast with the expected figures, the number of women with high-grade disease was exceptionally high. The correlation between Pap smear abnormalities and the real presence of cervical disease is not very favorable for low-grade cytological findings. In the ASCUS-LSIL Triage Study (ALTS), only 15% of women with ASC were diagnosed with significant histological lesions, and 50% of those with LSIL had abnormal cervical patterns visible via colposcopy.^9^

It is unreasonable to suppose that the cervical cancer screening deficiencies in the remote and impoverished regions of Brazil could be resolved in the short term. Cytology-based screening programs demand expensive and complex infrastructure. In addition, Pap smears do not yield results immediately, which is especially problematic in sparsely populated regions with severe transportation shortcomings, like most of the Brazilian Amazon region.

As reported and well discussed in previous Indian, African and even Brazilian reports, VIA might be an acceptable alternative to cytology in low-resource settings with high incidence of cervical neoplasia, considering the logistic and economic circumstances usually encountered in such regions. This is certainly the case in the geographic region where the present study was conducted. For instance, as advocated by Sankaranarayanan et al., VIA allows diagnosis and treatment of cervical abnormalities on a single occasion. This would clearly help overcome one of the main problems in the Amazon region: the huge distances between settlements and between primary healthcare units. VIA has also been reported to have an acceptable positive predictive value in populations that are highly affected by cervical disease, which is certainly the case in Cruzeiro do Sul.^12-16^ In the present study, the cervix of the patients was examined using acetic acid and the naked eye, and this increased the number of cervical abnormalities diagnosed by 25.5%. However, this examination should not be named VIA, because this procedure involves a standard routine, i.e. usage of 5% acetic acid and proper personnel training. On the other hand, there is an Atlas dedicated to VIA interpretation.^17^

## CONCLUSIONS

The results from the present study allow the conclusion that, in the Brazilian Amazon region, it is very likely that the official figures for the incidence and prevalence of cervical disease are underestimated. The large numbers of CIN and cancer cases that were detected might well reflect the ineffective screening in the region, and properly planned and structured public interventions might increase cervical cancer detection rates in such underserved geographical areas. According to data gathered by other authors, VIA might be a highly acceptable option for the screening of cervical abnormalities among the female population in the Amazon region.
